# Contact tracing evaluation for COVID-19 transmission in the different movement levels of a rural college town in the USA

**DOI:** 10.1038/s41598-021-83722-y

**Published:** 2021-03-01

**Authors:** Sifat A. Moon, Caterina M. Scoglio

**Affiliations:** Department of Electrical & Computer Engineering, Kansas State University, Manhattan, KS USA

**Keywords:** Computational models, Network topology, Computational science

## Abstract

Contact tracing can play a key role in controlling human-to-human transmission of a highly contagious disease such as COVID-19. We investigate the benefits and costs of contact tracing in the COVID-19 transmission. We estimate two unknown epidemic model parameters (basic reproductive number $R_0$ and confirmed rate $\delta _2$) by using confirmed case data. We model contact tracing in a two-layer network model. The two-layer network is composed by the contact network in the first layer and the tracing network in the second layer. In terms of benefits, simulation results show that increasing the fraction of traced contacts decreases the size of the epidemic. For example, tracing $25\%$ of the contacts is enough for any reopening scenario to reduce the number of confirmed cases by half. Considering the act of quarantining susceptible households as the contact tracing cost, we have observed an interesting phenomenon. The number of quarantined susceptible people increases with the increase of tracing because each individual confirmed case is mentioning more contacts. However, after reaching a maximum point, the number of quarantined susceptible people starts to decrease with the increase of tracing because the increment of the mentioned contacts is balanced by a reduced number of confirmed cases. The goal of this research is to assess the effectiveness of contact tracing for the containment of COVID-19 spreading in the different movement levels of a rural college town in the USA. Our research model is designed to be flexible and therefore, can be used to other geographic locations.

## Introduction

COVID-19 has affected the lives of billions of people in 2019–2020. The COVID-19 disease is caused by severe acute respiratory syndrome coronavirus 2 (SARS-CoV-2) and has caused a global health emergency. The world health organization (WHO) declared it as a Public Health Emergency of International Concern on January 30, 2020^1^. The number of confirmed reported cases by SARS-CoV-2 has been rising. On May 31, 2020, worldwide there were 5,939,234 laboratory-confirmed cases with 367,255 deaths^2^.

Many countries issued a pandemic lockdown to slow down the spreading of COVID-19. In the United States, a “Stay-At-Home” order was issued in many states. However, those pandemic lockdowns have a massive impact on the economy. All the States of the USA started reopening gradually from early May. Understanding the impact of mitigation strategies on the spreading dynamic of COVID-19 during the reopening phase of the USA is essential. In this work, we assess the impact of contact tracing by using an individual-based network model under four reopening scenarios: $25\%$ reopening, $50\%$ reopening, $75\%$ reopening, and $100\%$ reopening (no restriction).

Individual-based contact-network models are a powerful tool to model COVID-19 spreading due to its person-to-person spreading nature. In this work, we develop an individual-based network model for a college town, Manhattan, KS, where households represent nodes of the network. We select Manhattan, KS, as our study area, since it is a typical college town in a rural region of Kansas, the home of Kansas State University. There are 20,439 occupied households in Manhattan, KS, according to census 2018^3^. The connections between two individual households represent the contact probabilities between the members of the households. To develop the contact network, we consider age-stratification and use Google COVID-19 community mobility reports^4^. The individual-based approach provides the flexibility to observe the local dynamic at the individual level. It also allows us to include a mitigation strategy in the model at the individual level, such as contact tracing.

To design an epidemic model for COVID-19 is challenging, as many epidemic features of the disease are yet to be investigated, such as, for example, the transmission rate, the pre-symptomatic transmission rate, and the percentage of the asymptomatic population. These uncertain characteristics make epidemic modeling challenging as the outcomes of the model are sensitive to the assumption made on the uncertainties. Therefore, we use a simple epidemic model with five compartments—susceptible-exposed-infected-confirmed-removed (SEICR)—capable of imitating the COVID-19 transmission and flexible enough to cope with new information. This model has only two unknown parameters: the basic reproductive number $R_0$, and the confirmed case rate or reporting rate $\delta _2$. An analytical/numerical approach to the computation of $R_0$ can be found in Barril et al.^5^ and Breda et al.^6^, respectively. We use confirmed COVID-19 cases from March 25, 2020 to May 4, 2020 in Manhattan, KS as data, and estimate the unknown parameters from data. We use this period to estimate $R_0$ as there was no reopening in Manhattan, KS; therefore, the contact network was the same thorough the whole time. The other parameters are taken from the literature. In the spreading of COVID-19, there are pre-symptomatic and asymptomatic cases that do not show any sign of illness^7^. Besides, there is a strong possibility that infected cases not detected exist. In our epidemic model, we have considered those unreported cases. We assume that a confirmed COVID-19 patient cannot spread the disease anymore except in his/her own household.

Since a vaccine is not available for COVID-19, contact tracing is a key mitigation strategy to control the spreading of COVID-19. Contact tracing is a mitigation strategy that aims at identifying people who may have come into contact with a patient. This mitigation strategy prevents further spreading by quarantine of exposed people. The public health personnel have used contact tracing as a tool to control disease-spreading for a long time^8^. We implement two approaches of the contact tracing strategy through a two-layer network model with two modified SEICR epidemic models. In the first contact tracing approach, we consider all the traced contacts of a confirmed case will be quarantined, which follows the CDC contact tracing guidance for COVID-19 (October 21, 2020)^9^. In the second contact tracing approach, we consider only the tested positive traced contacts of a confirmed case will be isolated. We propose two quarantine approach to compare their effectiveness. This research finds that quarantine all the traced contacts is always effective than quarantine only test positive traced contacts. Feasibility of contact tracing to control COVID-19 spreading was analyzed using a branching process stochastic simulation for three basic reproductive numbers $R_0=1.5, 2.5$, and 3.5^10^. The authors find that sufficient contact tracing with quarantine can control a new outbreak of COVID-19. They mostly focus on the question of how much contacts need to be traced to control an epidemic for the three levels of basic reproductive number. However, this article neither explored the effectiveness of contact tracing for a specific location, nor investigated the cost of contact tracing.

In this research, we develop an individual-based network framework to assess the impact of contact-tracing in the reopening process in a college town of Kansas. To analyze the cost of contact-tracing represented by the number of quarantined susceptible people, we develop a contact network and estimate the basic reproductive number $R_0$ and confirmed rate (infected to laboratory-confirmed transition) from observed confirmed case data in Manhattan KS. We use our individual-based network model and the estimated parameters to run simulations of COVID-19 transmission. We use our framework to understand the spreading of COVID-19 and assess the contact-tracing strategy in the different reopening situations and different levels of tracing contacts.

Summarizing, the main contributions of this paper are the following:

- A novel individual-level network-based epidemic model to assess the impact of contact tracing.
- A thorough investigation of costs and benefits of contact-tracing in the reopening process in a college town of Kansas.

The individual-based network model is developed to represent the heterogeneity in people mixing. Our individual-based network epidemic model is general and flexible. It can be used to estimate, and model contact-tracing for COVID-19 in any location. It can also be used for any other disease that has a similar spreading mechanism like COVID-19.

## Results

### Individual-based contact network model

We use demographic data to develop an individual-based contact network model capable of representing the heterogeneous social mixing. Our network has *N* nodes and *L* links. In this network, each node represents one occupied household, a link between two households represents the contact probability between members of these households. The system has a total population of *p* individuals, distributed randomly into the *N* occupied households according to five social characteristics: age, average household sizes, family households, couple, living-alone^3^. We maintain the average household sizes, number of family households, number of couples, and number of living-alone households. Besides, a person under 18 years old is always assigned in a house with at least one adult person. To develop this network, we consider five age-ranges: under 18, 18–24, 25–34, 35–59, and over 60. Each age-range has $p_i$ people, where $i \in \{1,2,3,4,5\}$. This model considers large shared living spaces (for example, dorms) as a set of households with 4–8 students in each household.

After assigning the people, an age-specific network is developed for each age range and a random mixing network for all ages. Then a combination of the six networks provides the full network. A full network represents a contact network for a typical situation. The configuration network model^11^ is used to develop age-specific networks and the random mixing network (details are given in the “Materials and methods”). According to census 2018, Manhattan, KS has *p* = 55,489 people and *N* = 20 439 occupied households^3^.

**Figure 1:**
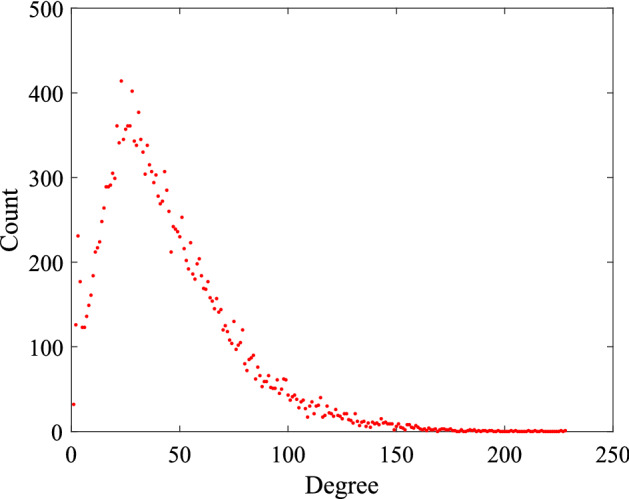
Degree distribution of the full network. In the network, households are at the node level. The network has 20,439 nodes and 445,350 edges. The average degree of this network is 43.647. The maximum degree in the network is 227.

Adjacency matrix for the full network $A_f$ is a summation of six adjacency matrices: $A_f=\sum _{i=1}^5{A_i} +A_r$. Here, $A_i$ is the adjacency matrix for the age-specific network *i*, and $A_r$ is the adjacency matrix for the random mixing network. Age-specific networks and the random mixing network are unweighted and undirected. However, the full network is a weighted and undirected network. The full network for Manhattan (KS) has 445,350 edges. The average node degree for an individual household in the full-network is 43.647, and for an individual person is 16.0518 (which is consistent with^12^). The degree distribution is presented in Fig. 1. The networks are available at https://doi.org/10.7910/DVN/3IM82E.

The full network is a contact network in the normal situation; we modify it to represent the contact network in the pandemic lockdown; we name it limited network. Manhattan, KS, is the home of Kansas State University. Most of the people living in Manhattan, KS, are closely related to Kansas State University, which halted its in-person activities from early March 2020 to August 17, 2020. Besides, Manhattan, KS was under the “Stay-At-Home” order from March 27, 2020 to May 4, 2020^13^. To represent this unusual situation, the full network is modified to a limited network version. As the educational institute was closed, we randomly reduce $90\%$ links from the age-specific networks for the age-ranges under 18, and $18-24$. The Google COVID-19 community mobility reports provide a percentage of movement changes in different places (for example, workplaces, recreational areas, parks)^4^. We reduced $40\%$ links randomly from the age-specific networks for 25–34, and 35–59 age-ranges for the movement changes in the workplaces^4^. The number of links in the limited network is 155,762. The limited network is available at https://doi.org/10.7910/DVN/3IM82E.

### Epidemic model

**Figure 2:**
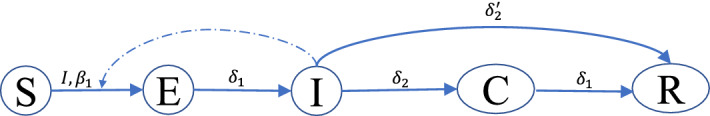
Node transition diagram of the susceptible-exposed-infected-confirmed (SEICR) epidemic model. This model has five compartments: susceptible (*S*), exposed (*E*), infected (*I*), confirmed (*C*), and removed (*R*) compartments. The SEICR model has five transitions (presented by solid lines): $S\rightarrow E$ (edge-based), $E \rightarrow I$ (nodal), $I \rightarrow C$ (nodal), $C \rightarrow R$ (nodal), and $I \rightarrow R$ (nodal). The infected (I) compartment is the influencer compartment of the edge-based $S\rightarrow E$ transition. The dashed line presents the influence of the *I* compartment on the $S\rightarrow E$ transition. We estimate $R_0$ and $\delta _2$ transition rate from data. We deduce $\beta _1$ from $R_0$.

We design a susceptible-exposed-infected-confirmed-removed (SEICR) epidemic scheme to simulate the spreading of COVID-19 (Fig. 2). This model has five compartments: susceptible *S*, exposed *E*, infected *I*, confirmed *C*, and removed *R*. A susceptible node is a node that is not infected yet. An exposed node is a node infected by the disease, but the viremia level is deficient that it cannot infect other nodes. An infected node is infectious, and it can infect other nodes. In this model, an infected node can be symptomatic, asymptomatic, or presymptomatic. A confirmed node is a laboratory-confirmed COVID-19 case. A removed node can be recovered or dead. The SEICR model has five transitions, which are divided into two categories: edge-based ($S \rightarrow E$), and nodal ($E \rightarrow I$; $I \rightarrow C$; $C \rightarrow R$; $I \rightarrow R$) transitions^14,15^.

An edge-based transition of a node depends on the state of its contacting nodes or neighbors in the contact network with its own state. A nodal transition of a node only depends on the own state. Each edge-based transition has an influencer compartment. A transition from susceptible to exposed ($S \rightarrow E$) of a susceptible node depends on the infected neighbors of that node. Therefore it is an edge-based transition, and the infected compartment is the influencer compartment of this transition. In this work, we are using the term ‘neighbors of a node *k*’ for the nodes, which have the shortest path length 1 from the node *k*. The transition rate of the susceptible to exposed ($S \rightarrow E$) transition of a node *k* is $\beta _1\sum \nolimits _l^N{A_c(k,l)I_l}$, here, $\beta _1$ is the transmission rate from one infected node to one susceptible node, $A_c$ is the adjacency matrix of the contact network, if *l* node is infected then $I_l=1$ otherwise $I_l=0$, and $\sum \nolimits _l^N{A_c(k,l)I_l}$ is the number of infected neighbors of the node *k*. The transition rate for the transition exposed to infected ($E \rightarrow I$) is $\delta _1$. The confirmed rate of an infected person is $\delta _2$. We consider that a laboratory-confirmed case will be isolated and cannot spread the disease outside of his household anymore. The unknown COVID-19 cases will move from infected to removed with a rate $\delta _2^{'}$. We add another transition $C \rightarrow R$ with rate $\delta _1$, this transition does not have any significance in the disease spreading. All the transition rates are exponentially distributed with a constant average value (Table 1). A detail of the SEICR epidemic model is stated in Table 1.

**Table 1:** Description of the susceptible-exposed-infected-confirmed (SEICR) epidemic model.

States	Type	Transition	Average transition rate ($days^{-1}$)	Influencer	Source
*S* (Susceptible) *E* (Exposed) *I*(Infected) *C* (Confirmed)	Edge-based	$S \rightarrow E$	$\beta _1\sum \limits _l^N{A_c(k,l)I_l}$ here, $\beta _1=\frac{R_0\delta _2}{\langle d \rangle \langle w \rangle }$; $\langle d \rangle$ = average degree; $\langle w \rangle$ = average weight	Neighbors in state *I*	$R_0$ is estimated
	Nodal	$E \rightarrow I$	$\delta _1=\frac{1}{3}$	–	^16,17^
		$I \rightarrow C$	$\delta _2=\frac{1}{4.56}$	–	Estimated
		$C \rightarrow R$	$\delta _1=\frac{1}{3}$	–	Model
		$I \rightarrow R$	$\delta _2^{'} =0.66\delta _2$	–	^18^

#### Parameter estimation for the SEICR epidemic model

The SEICR model has two unknown parameters: basic reproductive number $R_0$, and confirmed or reporting rate $\delta _2$. To estimate the $R_0$ and $\delta _2$, we have used confirmed cases in Riley County (Kansas) from March 25, 2020 to May 4, 2020. In this period, Kansas State University was closed, and “Stay-At-Home” order was there. It is reasonable to use this time period to estimate $R_0$ as there was no reopening and the mobility was the same throughout the period in Manhattan, KS. For the simulation of this period, a limited network is used (explained in the “Materials and methods” section), which is a modified version of the Full network to simulate the particular situation under the “Stay-At-Home” order. We use approximate Bayesian computation based on sequential Monte Carlo sampling (ABS-SMC) approach to estimate $R_0$ and $\delta _2$^19,20^. Other parameters ($\delta _1$^16,17^, and $\delta _2^{'}$^18^) are taken from the literature.

The estimated value for $R_0$ is 0.55 ($95\% \text { confidence interval: } 0.522-0.564$) and for reporting rate $\delta _2$ is $\frac{1}{4.79}\text {day}^{-1}$ ($95\%$ confidence interval: $\frac{1}{4.89}-\frac{1}{4.74}\text {day}^{-1}$). These estimated values are specific for Manhattan, KS for the time from March 25, 2020 to May 4, 2020. The $R_0$ for different reopening scenarios is presented in the supplementary Fig. S1. We consider that some people will develop severe symptoms, and they will be reported as a confirmed case of COVID-19 sooner. However, some people will produce deficient symptoms, and may they will be tested later. Therefore, the estimated confirmed rate is an average of all possibilities.

**Figure 3:**
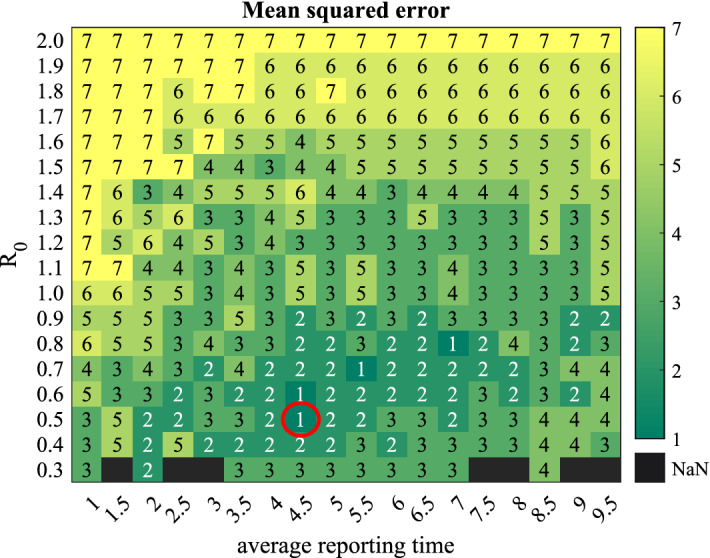
A sensitivity analysis. Mean-squared error (mse) between the time series of the total confirmed cases (or cumulative new cases per day) of March 25, 2020 to May 4, 2020 and simulated results for a different combination of basic reproductive number and average reporting time (in days). The light-colored boxes represent more mse than dark-colored boxes. The color boxes with number “1” means that mse ≤ 3, number “2” means that 3 < mse ≤ 10, number “3” means that 10 < mse ≤ 50, number “4” means that 50 < mse ≤100, number “5” means that $100<$mse $\le 500$, number “6” means that 500 < mse ≤ 1000, number “7” means that 1000 < mse. More than $80\%$ times epidemic dies out in the combinations of the black squares, and confirmed cases are less than 10. The minimum error combination is showing by the red circle. We estimate $R_0=0.55$ and average reporting time= 4.79 days.

A sensitivity analysis for $R_0$ and reporting time on the mean-squared error between confirmed cases data and simulated results is presented in Fig. 3.

#### Simulation for four different reopening scenarios

**Figure 4:**
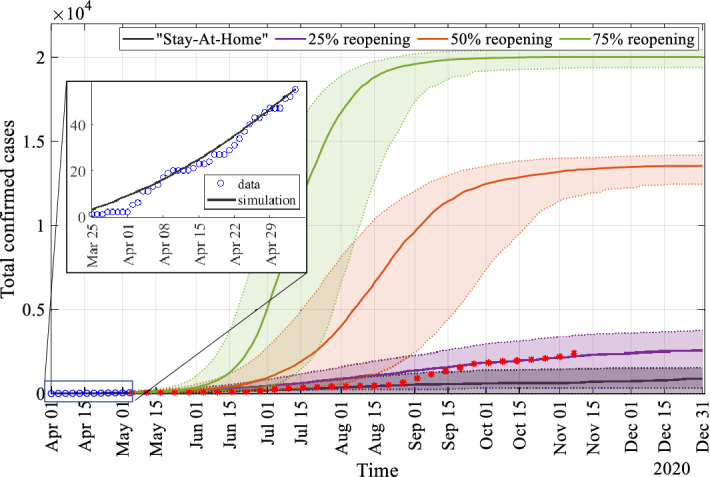
Total confirmed cases with time in the four reopening scenarios after ‘stay at home’ order lifted on May 4, 2020. Solid lines represent the median, and shaded regions represent interquartile range of the 1000 stochastic realizations. The blue circles in the zoom-in window present the total confirmed case data of the COVID-19 in Manhattan (Kansas) for the time period from March 25, 2020 to May 4, 2020. We have used this time period to estimate the basic reproductive number and the average confirmed time. The red stars are the total confirmed case data of the COVID-19 in Manhattan (Kansas) after May 5, 2020.

We simulate the total confirmed cases (or cumulative new cases per day) for eight months: from May to December using the SEICR epidemic model with the estimated parameters. To simulate, we assume that there is no change except reopening from pandemic lockdown. We are presenting four reopening situations: “Stay-At-Home” is still there or no reopening, $25\%$ reopening, $50\%$ reopening, and $75\%$ reopening. Kansas has started to reopen step by step after May 4, 2020. We use the limited network to simulate from March 25, 2020 to May 4, 2020; then, we change the network concerning the reopening situation. For example, in a $25\%$ reopening situation, $25\%$ of the reduced movement will start again; to model it, we add $25\%$ missing links randomly (which are present in the full network but not in the limited network). We preserve the states of each node at May 4, 2020 in the network then use it as the initial condition for the simulation for the reopening situation (from May 4, 2020 to July 1, 2020). Figure 4 is showing the medians (solid lines) and interquartile ranges (shaded regions) of the total confirmed cases of the 1000 stochastic realizations of the four reopening scenarios. The zoom-in window in Fig. 4 shows the time period when data was used to estimate the parameters of the epidemic model.

### Contact tracing

Contact tracing is a key mitigation strategy to control the spreading of COVID-19. To implement contact tracing, we modify the basic SEICR epidemic model and propose a two-layer network model. In the implementation of the contact tracing, we follow the CDC’s guidance for contact tracing^9^.

#### Two-layer individual-based network model

**Figure 5:**
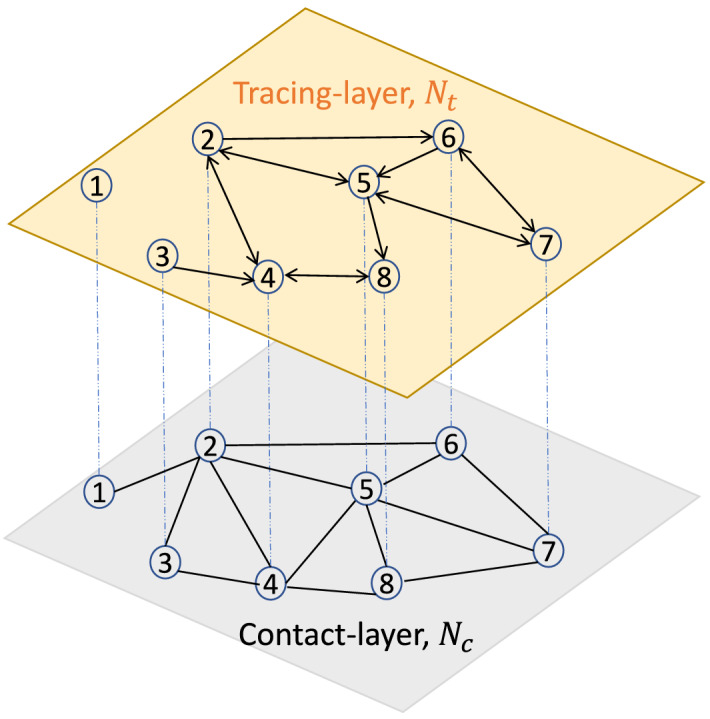
Two-layer network model: contact-layer $N_C$, and tracing-layer $N_t$. In this example, $50\%$ of contacts of each node is traced. For example, node 4 has four neighbors in the contact-layer (2, 3, 5, 8); however, two neighbors in the tracing-layer (2, 8). Node 7 has three neighbors in the contact-layer (6, 5, 8); however, two neighbors in the tracing-layer (6, 5). Node 8 has three neighbors in the contact-layer (4, 5, 7); however, one neighbor in the tracing-layer (4).

This work implements contact tracing in a two-layer network model: the contact network is in the first layer, and the tracing network is in the second layer (Fig. 5). We will call the first layer as the contact-layer and second layer as the tracing-layer in the rest of the paper. In the $t\%$-tracing-layer, $t\%$ of links of each node in the contact-layer are preserved randomly. To form a $t\%$-tracing-layer, at first, we generate a random number *r* from *U*(0, 1) for each link from a node *i*; then keep the link in the tracing-layer if $r\le 0.01t$. A $50\%$ tracing-layer is presented in Fig. 5. Although the contact-layer is an undirected network, however, the tracing-layer is a directed network. In the directed tracing-layer, a neighboring node of a node *i* has a distance one from node *i*. The neighbors of a confirmed (C) node in the tracing-layer will be tested and quarantined.

#### Epidemic scheme for contact tracing

For the contact tracing mitigation strategy, we consider two approaches for quarantine: I) all the neighbors of a confirmed case in the tracing-layer will be quarantined, and II) only infected neighbors of a confirmed case in the tracing-layer will be isolated. For the case I, we propose the SEICQ1 epidemic model, and for case II, we propose the SEICQ2 epidemic model (details are given in the “Materials and methods”).

#### Impact of contact tracing

**Figure 6:**
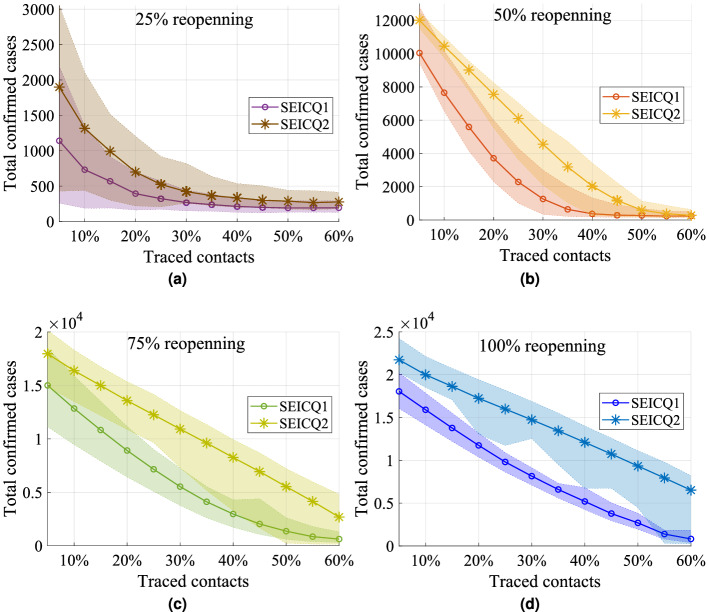
Impact of contact tracing. Total reported cases in eight months after ‘Stay-At-Home order’ lifted for different movement restrictions scenarios. Contact tracing is applied after May 4, 2020. This figure is showing the median (solid lines) and interquartile range (shaded regions) value of 1000 stochastic realizations.

Contact tracing can minimize the effect of the reopening process and control the spreading of COVID-19. We apply contact tracing after May 4, 2020 in Manhattan, KS. The plot of total confirmed cases on December 31, 2020 is presented in Fig. 6 for four reopening scenarios : $25\%$ reopening, $50\%$ reopening, $75\%$ reopening, and $100\%$ reopening for the different levels of contact tracing. The solid lines in Fig. 6 represent the median, and shaded regions represent the interquartile range of the 1000 stochastic realizations for the SEICQ1 and SEICQ2 model.

The difference between SEICQ1 and SEICQ2 is that SEICQ1 quarantines susceptible, exposed, and infected neighbors of a confirmed case in the tracing-layer; however, SEICQ2 isolates only the infected neighbors of a confirmed case in the tracing-layer. The SEICQ1 model is always efficient than the SEICQ2 model to control the COVID-19 spreading. However, both approaches can reduce the number of confirmed cases, even in the $100\%$ reopening situation. For any reopening situations, tracing more than $55\%$ of the contacts in the SEICQ1 can reduce the median of the 1000 stochastic realizations of the confirmed cases more than $90\%$, and in the SEICQ2 can reduce the median of the 1000 stochastic realizations of the confirmed cases more than $66\%$ on December 31, 2020, with compare to no-contact-tracing (SEICR model) (Table 2).

**Table 2:** Percentage of reduction of the total confirmed cases in eight months after May 4, 2020, in the four reopening scenarios for the two contact tracing mitigation approaches.

Traced contacts	Percentage of reduction in the total confirmed cases							
	SEICQ1				SEICQ2			
	$25\%$ reopening	$50\%$ reopening	$75\%$ reopening	$100\%$ reopening	$25\%$ reopening	$50\%$ reopening	$75\%$ reopening	$100\%$ reopening
$5\%$	55.38	25.90	25.06	24.52	25.70	11.36	10.28	9.10
$10\%$	71.37	43.50	35.87	33.5	48.54	22.84	18.20	16.40
$15\%$	77.73	58.7	45.91	42.39	61.14	33.44	25.14	22.08
$20\%$	84.60	72.60	55.49	42.39	72.64	44.24	32.21	27.90
$25\%$	87.35	83.14	64.26	58.98	79.51	55.01	38.80	33.14
$30\%$	89.52	90.67	72.37	65.90	83.47	66.36	45.47	38.39
$35\%$	90.65	95.29	79.45	72.43	85.74	76.46	51.99	43.80
$40\%$	91.67	97.26	85.16	78.28	86.90	85.00	58.76	49.40
$45\%$	92.20	97.86	89.82	84.19	88.23	91.34	65.36	55.11
$50\%$	92.49	98.02	93.18	88.74	88.75	95.62	72.36	60.97
$55\%$	92.48	98.27	95.73	94.19	89.54	97.25	79.28	66.80
$60\%$	92.41	98.22	96.87	96.60	89.17	97.96	86.59	72.75

**Figure 7:**
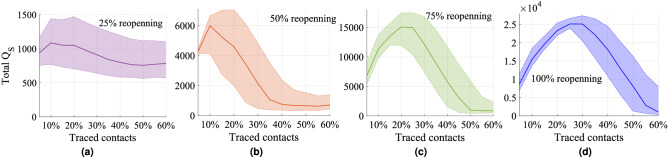
The total number of quarantined susceptible households in eight months after May 4, 2020, for the SEICQ1 epidemic model for the four reopening scenarios with different tracing levels. This figure is showing the median (solid lines) and interquartile range (shaded regions) of 1000 stochastic realizations.

The SEICQ1 model can reduce the reported cases further compared to SEICQ2 for the same amount of contact tracing (Fig. 6). However, the SEICQ1 model has a drawback; it quarantines susceptible persons. The number of total quarantined susceptible households in the simulation time period for different amounts of traced contacts for the SEICQ1 model is presented in Fig. 7 and Table 3. The quarantined susceptible households increase with the increase of tracing; however, after tracing a certain percentage ($t_p\%$) of contacts, the quarantined susceptible households start to decrease with the increase of tracing (Fig. 7). If we consider quarantined susceptible households are the cost of SEICQ1 model, then it is cost-effective to trace contacts of the confirmed cases more than $t_p\%$; which is $10\%$ for $25\%$ reopening, $10\%$ for $50\%$ reopening, $20\%$ for $75\%$ reopening, and $25\%$ for $100\%$ reopening (Table 3). The reason for decreasing the number of quarantined households with the increasing of contact-tracing after the maximum value is the smaller number of the infected cases. Although each confirmed case will give a long list of possible contacts, this effect will be balanced out by a decreasing number of the confirmed cases (supplementary Fig. S2–S5).

**Table 3:** Total quarantined susceptible households in eight months after May 4, 2020, in the SEICQ1 epidemic model for the four reopening scenarios.

Traced contacts	SEICQ1							
	$25\%$ reopening		$50\%$ reopening		$75\%$ reopening		$100\%$ reopening	
	Total confirmed cases (median)	Total quarantined Susceptible households (median)	Total confirmed cases (median)	Total quarantined Susceptible households (median)	Total confirmed cases (median)	Total quarantined Susceptible households (median)	Total confirmed cases (median)	Total quarantined Susceptible households (median)
$5\%$	1141	941	10039	4278	15,005	6796	18048	8727
$10\%$	732	1086	7653	5997	12,840	11,329	15,899	15,486
$15\%$	569	1052	5594	5231	10,829	13,515	13,775	19,706
$20\%$	393	1051	3712	4582	8911	15,071	11,740	23,355
$25\%$	323	978	2284	3401	7156	14,919	9807	25,150
$30\%$	268	911	1263	2124	5531	12,120	8151	25,122
$35\%$	239	840	638	1053	4113	8938	6589	22,120
$40\%$	213	800	370	748	2970	5791	5193	18,069
$45\%$	199	765	290	667	2039	3050	3780	12,883
$50\%$	192	757	267	659	1365	998	2693	8052
$55\%$	192	772	233	622	853	872	1386	2870
$60\%$	194	784	241	650	625	856	813	1037

## Discussion

This research studies contact tracing as a key mitigation strategy to control the COVID-19 transmission in the reopening process of a college town in the rural region of the USA. Therefore, we propose a general framework to develop an individual-based contact network epidemic model to estimate parameters and implement contact tracing. This model is used to estimate the basic reproductive number ($R_0$) and confirmed rate ($\delta _2$) in Manhattan, KS, for the COVID-19 spreading. The outcomes of this research are valuable to understand the effectiveness of the contact-tracing strategy in the different scenarios of the COVID-19 spreading. Furthermore, this framework is generic enough to use any locations and for other diseases as well.

The individual-based network model represents the heterogeneous mixing nature of a population. To investigate transmission at the individual level, we develop an individual-based contact network model where households are presented by network nodes. The contact network is a combination of five age-specific networks and one random-mixing network; this approach allows us to change an age-specific network according to any change in the society (for example, summer break, pandemic lockdown). The pandemic lockdown reduces the contacts mostly among the people who are students. Therefore, age-specific networks for under 18 and 18–24 are changed mostly. Pandemic lockdown also affects people in 25–34, 35–59 age-ranges. We propose a ‘full network’ to represent the usual situation; then, we modify the age-specific networks of the full network according to Google COVID-19 community mobility reports^4^ to represent pandemic lockdown . The modified network is the limited network, a reduced version of the full network. The average degree of the full network is 43.647 for Manhattan, KS which means that each household has probable direct connections with an average of 43.647 households. The full network is connected and provides an approximation of the contact network at the household level, which is useful for doing the simulation anonymously.

We propose a susceptible-exposed-infected-confirmed-removed (SEICR) epidemic model in the limited network to simulate COVID-19 transmission from March 25, 2020 to May 4, 2020. We estimate the unknown parameters of the SEICR model for the Manhattan, KS, using approximate Bayesian computation based on sequential Monte Carlo sampling. We use confirmed cases as an observed data set. Designing an optimal epidemic model to simulate epidemic spreading is essential. However, it is challenging to design an epidemic model for COVID-19 spreading with limited knowledge; understanding the spreading of COVID-19 needs more investigation. Concerning the unclear role of immunity, we assume that the immunity of a recovered COVID-19 patient is not going to fade in the short period analyzed in our simulations. In addition, we assume that a tested positive person is responsible enough to stay in isolation. However, it is important to keep the model simple, since the data available to estimate parameters is limited. Therefore, we propose a simple but dynamic and flexible epidemic model to simulate COVID-19 spreading, which has only two unknown parameters. The model can easily cope with additional information that may be available in the future.

The estimated basic reproductive number is much smaller in Manhattan, KS (estimated $R_0 =0.55$) because of the ‘Stay at home’ order. In Manhattan, $51\%$ of people have age below 24 years, who get a chance to stay at home because of the online curriculum in educational institutions. However, the basic reproductive number will change when educational institutes start their in-person curriculum (in the $100\%$ reopening, the deduced $R_0$ is 2.0301). There are 301 college towns in the USA^21^, which have a similar population structure like Manhattan, KS. A practical contact tracing approach can help to control the epidemic in those college towns.

We implement contact tracing using a two-layer network model. We assess the impact of contact tracing in the four reopening scenarios: 25$\%$ reopening, 50$\%$ reopening, 75$\%$ reopening, and 100$\%$ reopening (or no restrictions). Reopening without vaccination is challenging. It is essential to access the efficacy of the contact tracing in the reopening path. Our investigation indicates that more than $50\%$ contact tracing can control the spreading of COVID-19 even in the $100\%$ reopening situation. The number of quarantined susceptible people increases with the increase of traced contacts, however after a certain amount of tracing ($t_p\%$), the number of quarantined susceptible people decreases with the increases of the traced contacts. We consider that quarantined susceptible people represent the cost of SEICQ1 contact tracing model. Therefore it is cost-effective to trace more than $t_p\%$ contacts of a confirmed case. Our research finds that $t_p$ increases with the increase in mobility (Table 3).

Our investigation indicates that a sufficient amount of contact tracing can reduce the impact of COVID-19 spreading in the reopening process of a location. At first, the quarantined susceptible people increase with the percentage of traced contacts, however after a certain amount of traced contacts, the quarantined susceptible people start to decrease with the increase in the percentage of traced contacts.

## Materials and methods

### Data

The study area of this research is a college town in the rural region of the USA: Manhattan, KS. We use two data sets to develop our model. The first dataset contains the sociodemographic information from the census 2018, and the second dataset contains the COVID-19 incidence data.

### Contact network

We use configuration network model to develop age-specific networks and the random network. The system has *N* occupied households and *p* people. The steps are:

Step 1: For each person *j* (here, $j\in 1,2,...,p$), contacts $c_j$ is assigned from a Gaussian distribution ${{\mathcal {N}}}(\mu , \sigma ^2)$. The mean $\mu$ of the Gaussian distributions are taken from the average number of daily contacts per person in each age-range^12,22,23^. The average daily contacts per person are given in Table 4. For an under 18-year-old person, the number of contacts is assigned randomly from the ${{\mathcal {N}}}(13.91, 6.95)$ distribution. For a person in 18–24 years age, the number of contacts is assigned randomly from the ${{\mathcal {N}}}(21.25, 10.62)$ distribution. For a person in 25–34 years age, the number of contacts is assigned randomly from the ${{\mathcal {N}}}(21.3, 10.65)$ distribution. For a person in 35–59 years age, the number of contacts is assigned from the ${{\mathcal {N}}}(20.912, 10.46)$ distribution. For an over 60-year-old person, the number of contacts is assigned randomly from the ${{\mathcal {N}}}(10.7, 5.35)$ distribution. In the random-mixing-network, the number of contacts is assigned randomly from the ${{\mathcal {N}}}(2, 1)$ distribution for a person *j*. The Gaussian or normal distribution is the distribution of real numbers; therefore, the number from the ${{\mathcal {N}}}(\mu , \sigma ^2)$ distribution is rounded to the closest integer.
Step 2: For each person *j*, contacts for its belonging household *k* is assigned by ($c_j- h_k-1$). Here, $c_j$ is the number of contacts for a person *j*, $h_k$ is the household size or number of people of the household *k*, person *j* lives in the household *k*, $j=1,2,3......p$, and $k=1,2,3......N$.
Step 3: From the mixing patterns of different age-ranges, people have a strong tendency to meet people with their same age range (more than 80$\%$)^12,22,23^. Therefore, We keep the maximum number of contacts among the same age ranges and a small percentage for the other age ranges. The percentage of contacts in the same age-specific-network for each age-range is given in Table 4. Degree $d_{ki}$ of a node *k* in the age-specific network *i* is $s\%$ of ($c_j- h_k-1$), here, $s\%$ of average daily contacts of a person happens with the people of his same age-range.
Step 4: After assigning degree, $d_{ki}$ for *N* nodes or households, The configuration network model^11^ creates half-edges for each node, then chooses two nodes randomly and connect their half-edges to form a full edge^11^.

The population and network characteristics for the five age-specific networks for Manhattan, KS are given in Table 4.

**Table 4:** Properties of the Age-specific-networks of the Manhattan, KS.

Age-range	Under 18	18–24	25–34	35–59	over 60
Population	8074	20,378	9887	10,581	6567
Average daily contacts per person^12^	13.91	21.25	21.3	20.91	10.7
Average daily contacts with non-household members per person	12.00	20.00	19.98	19.00	7.05
$\%$ of neighbors in the same age-specific networks^22^	85.63	90.48	90.29	84.95	71.43
Number of edges in the age-specific networks	40,466	187,723	88,806	90,835	16,511

### Stochastic simulation

To do the simulation, we use GEMFsim; it is a stochastic simulator for the generalized epidemic modeling framework (GEMF), which was developed by the Network Science and Engineering (NetSE) group at Kansas State University^24^. The GEMFsim is a continuous-time, individual-based, numerical simulator for the GEMF-based processes^14^. The network and epidemic model is the input of the GEMFsim, and the time dynamic of each node state is the output. In GEMF, the joint state of all nodes follows a Markov process that arises from node-level transition. A node can change its state by moving from one compartment to another compartment through a transition. One assumption of the GEMF system is, all the events or transitions are independent Poisson processes with the constant rate; this assumption leads the system to a continuous-time Markov process. Initially, the simulation starts by setting two infected nodes randomly. The stochastic simulator GEMFsim is based on the Gillespie algorithm. The Gillespie algorithm can produce a statistically correct trajectory of a continuous-time Markov process.

### Epidemic model for contact tracing

The SEICQ1 model has eight compartments: susceptible (*S*), exposed (*E*), infected (*I*), confirmed (*C*), quarantined-susceptible ($Q_S$), quarantined-exposed ($Q_E$), quarantined-infected ($Q_I$), and removed (*R*). The SEICQ2 model has six compartments: susceptible (*S*), exposed (*E*), infected (*I*), confirmed (*C*), quarantined-infected ($Q_I$), and removed (*R*). The transitions $S\rightarrow E$, $E\rightarrow I$, $I\rightarrow C$, and $I\rightarrow R$ are the same as the base SEICR model.

**Figure 8:**
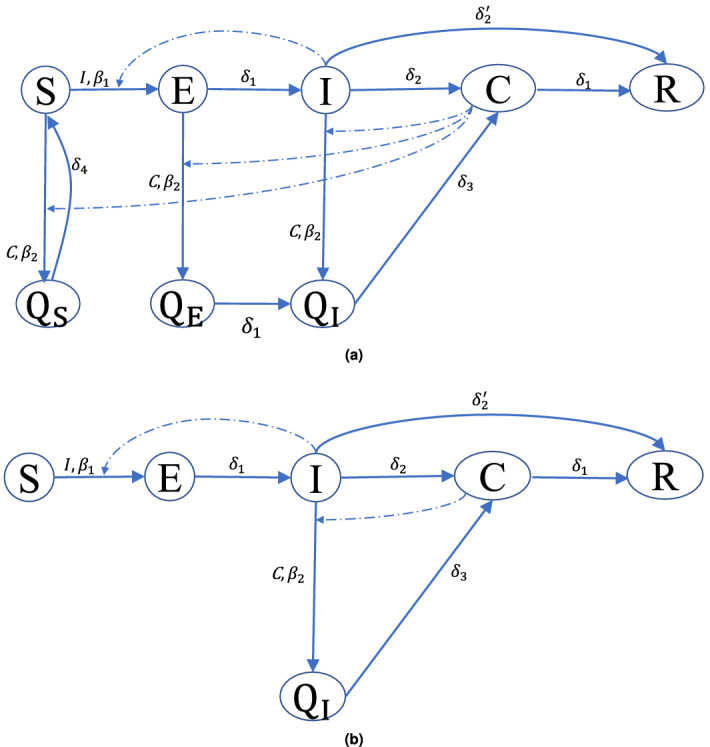
Node transition diagrams. (**a**) SEICQ1 epidemic model, (**b**) SEICQ2 epidemic model. The solid lines represent the node-level transitions, and the dashed lines represent the influence of the influencer compartment on an edge-based transition.

In the SEICQ1 model, neighbors (susceptible, exposed, and infected) of a confirmed node in the tracing-layer will be tested and quarantined. In the SEICQ1 model, susceptible, exposed, infected neighbors in the tracing-layer of a confirmed case will go to the quarantined-susceptible $Q_S$, quarantined-exposed $Q_E$, and quarantined-infected $Q_I$ states with rate $\beta _2$. The susceptible to quarantined-susceptible ($S \rightarrow Q_S$), exposed to quarantined-exposed ($E \rightarrow Q_E$), and infected to quarantined-infected ($I \rightarrow Q_I$) transitions are edge-based transitions and confirmed compartment is the influencer of these transitions. A COVID-19 positive neighbor of a confirmed node will go to the confirmed state immediately with $\delta _3$ rate, $Q_I \rightarrow C$ is a nodal transition. We model the transition rates $\beta _2$ and $\delta _3$ are much higher than the rate for the transition $C \rightarrow R$ to ensure that the neighbors of a confirmed node in the tracing-layer will move to the quarantined or confirmed state before the $C \rightarrow R$ event happens. For the simulation, we take $\beta _2=\delta _3=50\delta _1$. The SEICQ1 model is presented in Fig. 8a. A description of the 11 transitions of the SEICQ1 model is given in Table 5.

In the SEICQ2 model, neighbors of a confirmed node in the tracing-layer will be tested, and only infected neighbors will go to the quarantined-infected ($Q_I$) state immediately with rate $\beta _2$. The node transition diagram of the SEICQ2 model is given in Fig. 8b. A description of the 7 transitions of the SEICQ2 model is given in the Table 6.

**Table 5:** Description of the SEICQ1 epidemic model.

States	Type	Transition	Transition rate ($days^{-1}$)	Inducer	Source
*S* (Susceptible) *E* (Exposed) *I* (Infected) *C* (Confirmed) $Q_S$(Quarantined-Susceptible) $Q_E$(Quarantined-Exposed) $Q_I$(Quarantined-Infected) *R* (Removed)	Edge-based	$S \rightarrow E$	$\beta _1\sum \limits _l^N{A_c(k,l)I_l}$ here, $\beta _1=\frac{R_0\delta _2}{\langle d \rangle \langle w \rangle }$; $\langle d \rangle$ = average degree; $\langle w \rangle$ = average weight	Neighbors of state *I* in the contact-layer	$R_0$ is estimated
		$S \rightarrow Q_S$	$\beta _2\sum \limits _l{A_t(k,l)C_l}$ here, $\beta _2>> \delta _1$, we take $\beta _2=50\delta _1$	Neighbors of state *C* in the tracing-layer	Model
		$$E\rightarrow Q_E$$			
		$$I \rightarrow Q_I$$			
	Nodal	$E \rightarrow I$	$\delta _1=\frac{1}{3}$		^16,17^
		$Q_E \rightarrow Q_I$		–	
		$C \rightarrow R$	$\delta _1 =\frac{1}{3}$	–	Model
		$I \rightarrow C$	$\delta _2=\frac{1}{4.56}$	–	Estimated
		$I \rightarrow R$	$\delta _2^{'} =0.66\delta _2$	–	^18^
		$Q_I \rightarrow C$	$\delta _3>> \delta _1$, we take $\delta _3=50\delta _1$	–	Model
		$Q_S \rightarrow S$	$\delta _4= \frac{1}{14}$	–	^8^

**Table 6:** Description of the SEICQ2 epidemic model.

States	Type	Transition	Transition rate ($days^{-1}$)	Inducer	Source
*S* (Susceptible) *E* (Exposed) *I* (Infected) *C* (Confirmed) $Q_I$(Quarantined-Infected) *R* (Removed)	Edge-based	$S \rightarrow E$	$\beta _1\sum \limits _l^N{A_c(k,l)I_l}$ here, $\beta _1=\frac{R_0\delta _2}{\langle d \rangle \langle w \rangle }$; $\langle d \rangle$ = average degree; $\langle w \rangle$ = average weight	Neighbors of state *I* in the contact-layer	$R_0$ is estimated
	Nodal	$I \rightarrow Q_I$	$\beta _2\sum \limits _l{A_t(k,l)C_l}$ here, $\beta _2>> \delta _1$, we take $\beta _2=50\delta _1$	Neighbors of state *C* in the tracing-layer	Model
		$E \rightarrow I$	$\delta _1=\frac{1}{3}$		^16,17^
		$C \rightarrow R$	$\delta _1 =\frac{1}{3}$	–	Model
		$I \rightarrow C$	$\delta _2=\frac{1}{4.56}$	–	Estimated
		$I \rightarrow R$	$\delta _2^{'} =0.66\delta _2$	–	^18^
		$Q_I \rightarrow C$	$\delta _3>> \delta _1$, we take $\delta _3=50\delta _1$	–	Model

## Supplementary information

Supplementary Figures.

## Data Availability

The dataset and code used to perform this research is available from https://doi.org/10.7910/DVN/3IM82E. The authors are willing to provide further details upon request.

## References

[1] Sohrabi C, Alsafi Z, O'Neill N, Khan M, Kerwan A, Al-Jabir A, Iosifidis C, Agha R (2020). World health organization declares global emergency: a review of the 2019 novel coronavirus (covid-19). Int. J. Surg..

[2] World health organization. https://covid19.who.int/. Accessed 31 May 2020.

[3] U.S. Census Bureau (2018). Selected social characteristics in the united states. https://data.census.gov/cedsci/. Accessed 30 March 2020.

[4] Google covid-19 community mobility reports. https://www.google.com/covid19/mobility/. Accessed 31 May 2020.

[5] Barril C, Calsina À, Ripoll J (2018). A practical approach to r 0 in continuous-time ecological models. Math. Methods Appl. Sci..

[6] Breda D, Florian F, Ripoll J, Vermiglio R (2021). Efficient numerical computation of the basic reproduction number for structured populations. J. Comput. Appl. Math..

[7] Furukawa N, Brooks J, Sobel J (2020). Evidence supporting transmission of severe acute respiratory syndrome coronavirus 2 while presymptomatic or asymptomatic. Emerg. Infect. Dis..

[8] Centers for disease control and prevention. https://www.cdc.gov/coronavirus/2019-ncov/php/contact-tracing/contact-tracing-plan/overview.html. Accessed 31 May 2020.

[9] Centers for disease control and prevention. https://www.cdc.gov/coronavirus/2019-ncov/index.html. Accessed 31 May 2020.

[10] Hellewell J, Abbott S, Gimma A, Bosse NI, Jarvis CI, Russell TW, Munday JD, Kucharski AJ, Edmunds WJ, Sun F, Flasche S (2020). Feasibility of controlling COVID-19 outbreaks by isolation of cases and contacts. Lancet Glob. Health.

[11] Newman M (2018). Networks.

[12] Del Valle SY, Hyman JM, Hethcote HW, Eubank SG (2007). Mixing patterns between age groups in social networks. Soc. Netw..

[13] Riley county health department; local health order no.3 issued march 27,2020 ”stay at home”. https://www.rileycountyks.gov/DocumentCenter/View/18553/03-27-2020---STAY-AT-HOME-ORDER-FROM-LOCAL-HEALTH-OFFICER-pdf. Accessed 31 May 2020.

[14] Sahneh FD, Scoglio C, Van Mieghem P (2013). Generalized epidemic mean-field model for spreading processes over multilayer complex networks. IEEE/ACM Trans. Netw..

[15] Moon, S. A., Sahneh, F. D. & Scoglio, C. Generalized group-based epidemic model for spreading processes on networks: Ggroupem. arXiv preprint arXiv:1908.06057 (2019).

[16] Brett TS, Rohani P (2020). Transmission dynamics reveal the impracticality of covid-19 herd immunity strategies. Proc. Natl. Acad. Sci..

[17] Lauer, S. A. *et al.* The incubation period of coronavirus disease 2019 (covid-19) from publicly reported confirmed cases: estimation and application. *Annals of internal medicine* (2020). 10.7326/M20-0504 PMC7081172 32150748

[18] Covid-19 pandemic planning scenarios, centers for disease control and prevention. https://www.cdc.gov/coronavirus/2019-ncov/hcp/planning-scenarios.html. Accessed 20 Nov 2020.

[19] Toni T, Welch D, Strelkowa N, Ipsen A, Stumpf MP (2009). Approximate Bayesian computation scheme for parameter inference and model selection in dynamical systems. J. R. Soc. Interface.

[20] Moon SA, Cohnstaedt LW, McVey DS, Scoglio CM (2019). A spatio-temporal individual-based network framework for west nile virus in the usa: spreading pattern of west nile virus. PLoS Comput. Biol..

[21] Gumprecht, B. College towns in the united states: Table. *The Am. Coll. Town* **1**, (2008).

[22] Wallinga J, Teunis P, Kretzschmar M (2006). Using data on social contacts to estimate age-specific transmission parameters for respiratory-spread infectious agents. Am. J. Epidemiol..

[23] Read JM, Eames KT, Edmunds WJ (2008). Dynamic social networks and the implications for the spread of infectious disease. J. R. Soc. Interface.

[24] Sahneh FD, Vajdi A, Shakeri H, Fan F, Scoglio C (2017). Gemfsim: a stochastic simulator for the generalized epidemic modeling framework. J. Comput. Sci..

