# Supplementary material for: Contact tracing evaluation for COVID-19 transmission in the different movement levels of a rural college town in the USA
Source: Sci Rep. 2021 Mar 1;11:4891. doi: 10.1038/s41598-021-83722-y (PMC7921112; doi:10.1038/s41598-021-83722-y)
Supplement: Supplementary file 1 — Supplementary Figures. [file 41598_2021_83722_MOESM1_ESM.pdf]

Contact Tracing Evaluation for COVID-19 Transmission in the different movement levels of a Rural College Town in the USA

Sifat A. Moon[1,*] **and Caterina M. Scoglio**[1]

[1]Department of Electrical & Computer Engineering, Kansas State University, Manhattan, Kansas, United States of America
[*]sifatafroj@ksu.edu

1 R0 in the different reopening scenarios in the Manhattan, KS

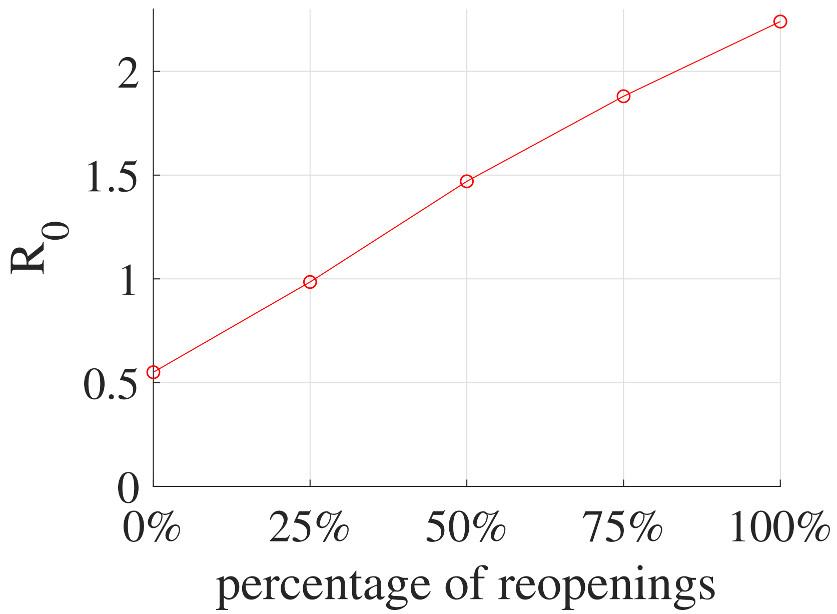

Figure S1. R_0 in different reopening scenarios for Manhattan, KS. Only R_0 in the 0% reopening is estimated from confirmed case data, and the other R_0 values are deduced from the network and simulation.

2 Analysis of quarantined susceptible households and total confirmed cases with time

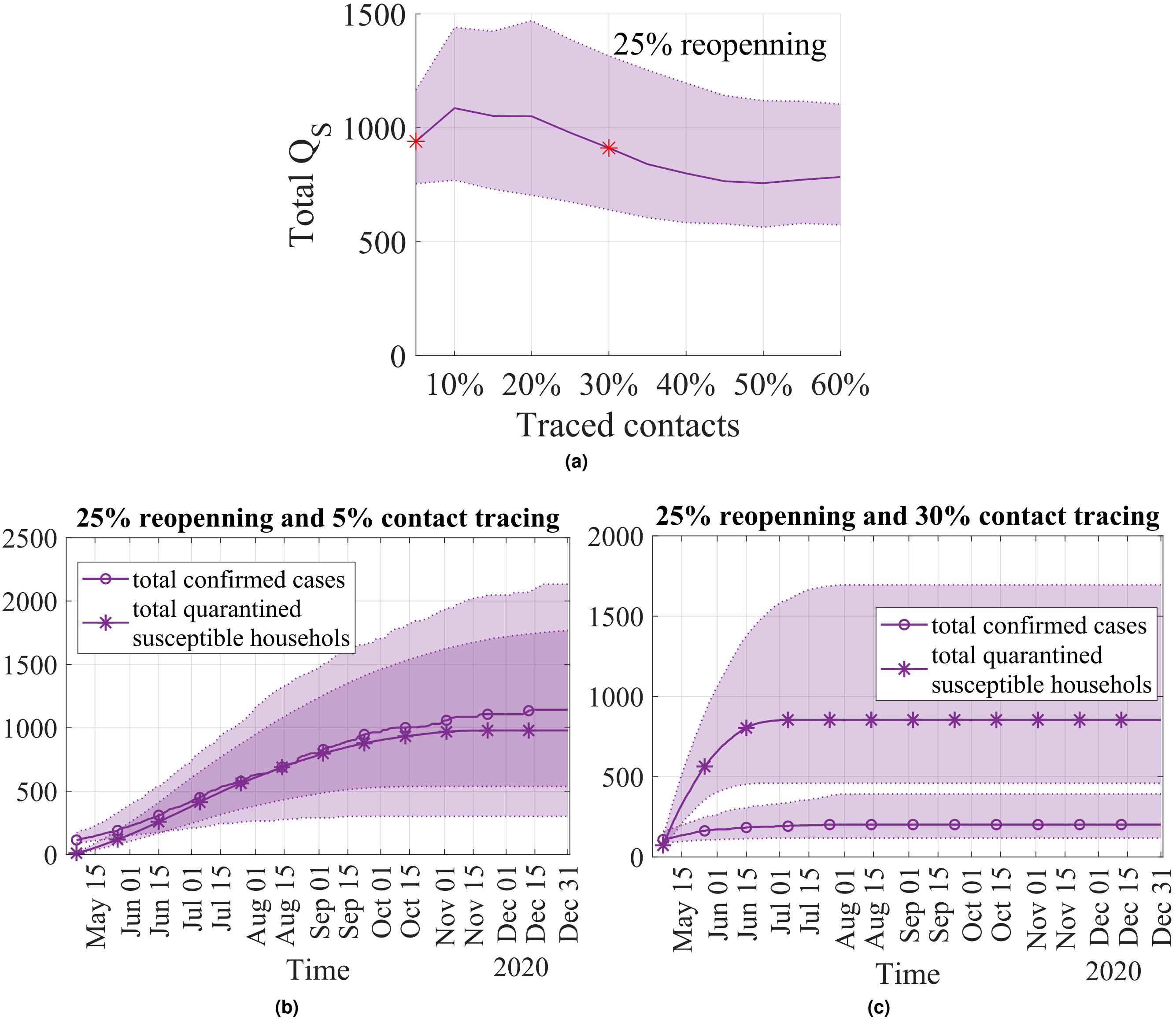

Figure S2. (a) The total number of quarantined susceptible households in eight months after May 4, 2020, for the SEICQ1 epidemic model for the 25% reopening scenarios with different tracing levels. (b) The total confirmed cases and the total quarantined susceptible households with time for the 5% contact tracing (for the first red star in the sub-figure (a)). (c) The total confirmed cases and the total quarantined susceptible households with time for the 30% contact tracing (for the second red star in the sub-figure (a)) This figure is showing the median (solid lines) and interquartile range (shaded regions) of 1000 stochastic realizations.

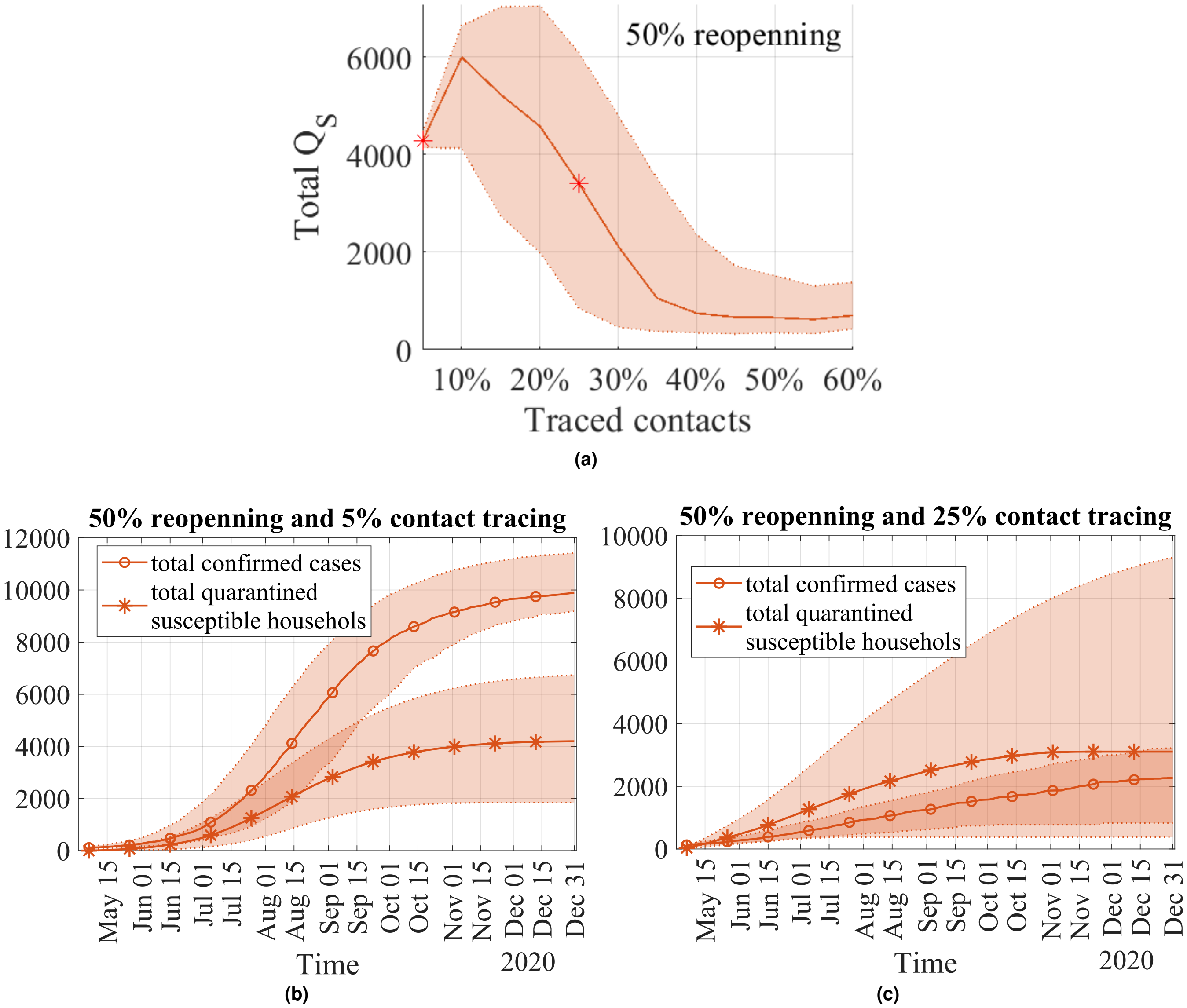

Figure S3. (a) The total number of quarantined susceptible households in eight months after May 4, 2020, for the SEICQ1 epidemic model for the 50% reopening scenarios with different tracing levels. (b) The total confirmed cases and the total quarantined susceptible households with time for the 5% contact tracing (for the first red star in the sub-figure (a)). (c) The total confirmed cases and the total quarantined susceptible households with time for the 25% contact tracing (for the second red star in the sub-figure (a)) This figure is showing the median (solid lines) and interquartile range (shaded regions) of 1000 stochastic realizations.

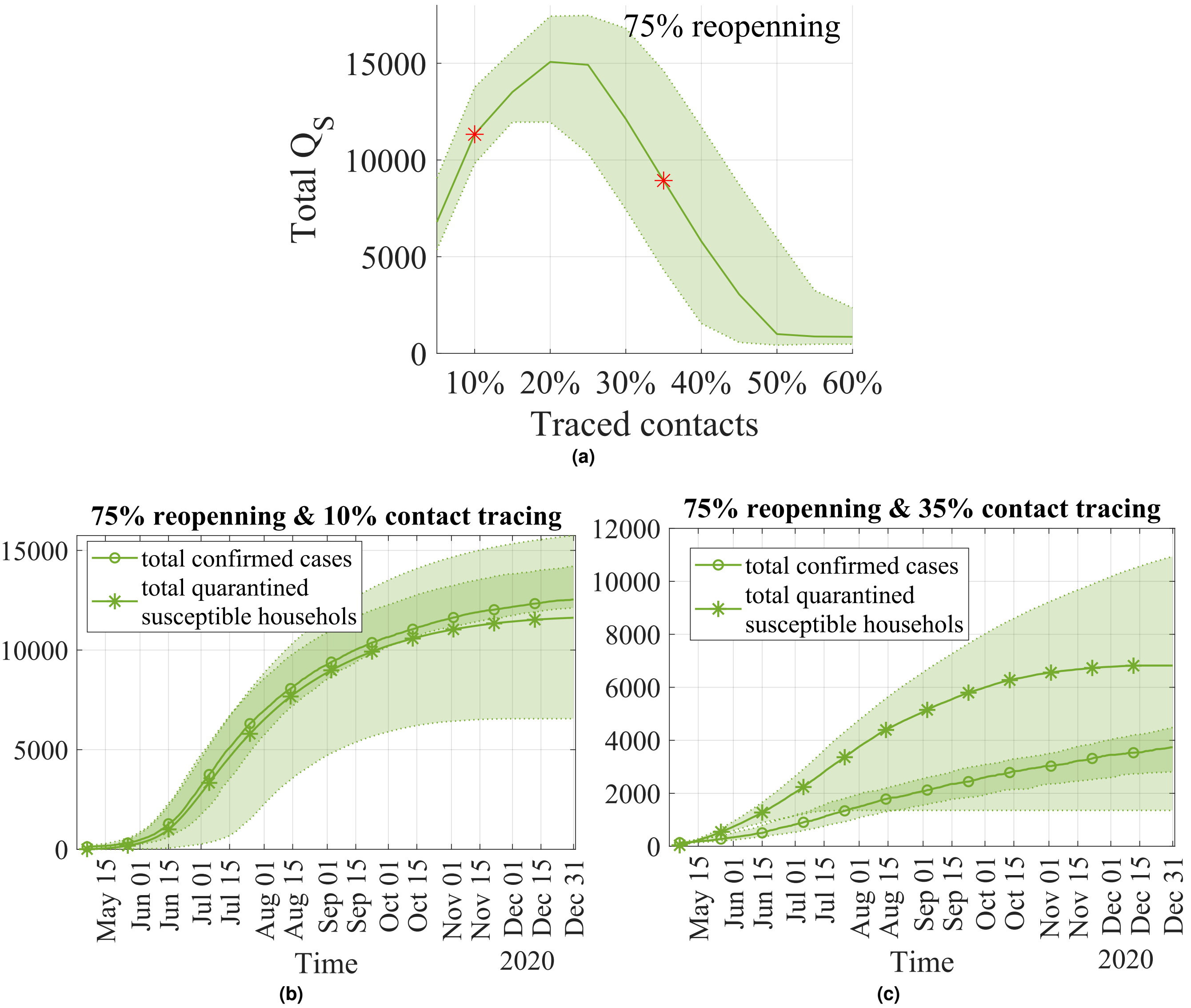

Figure S4. (a) The total number of quarantined susceptible households in eight months after May 4, 2020, for the SEICQ1 epidemic model for the 75% reopening scenarios with different tracing levels. (b) The total confirmed cases and the total quarantined susceptible households with time for the 10% contact tracing (for the first red star in the sub-figure (a)). (c) The total confirmed cases and the total quarantined susceptible households with time for the 35% contact tracing (for the second red star in the sub-figure (a)) This figure is showing the median (solid lines) and interquartile range (shaded regions) of 1000 stochastic realizations.

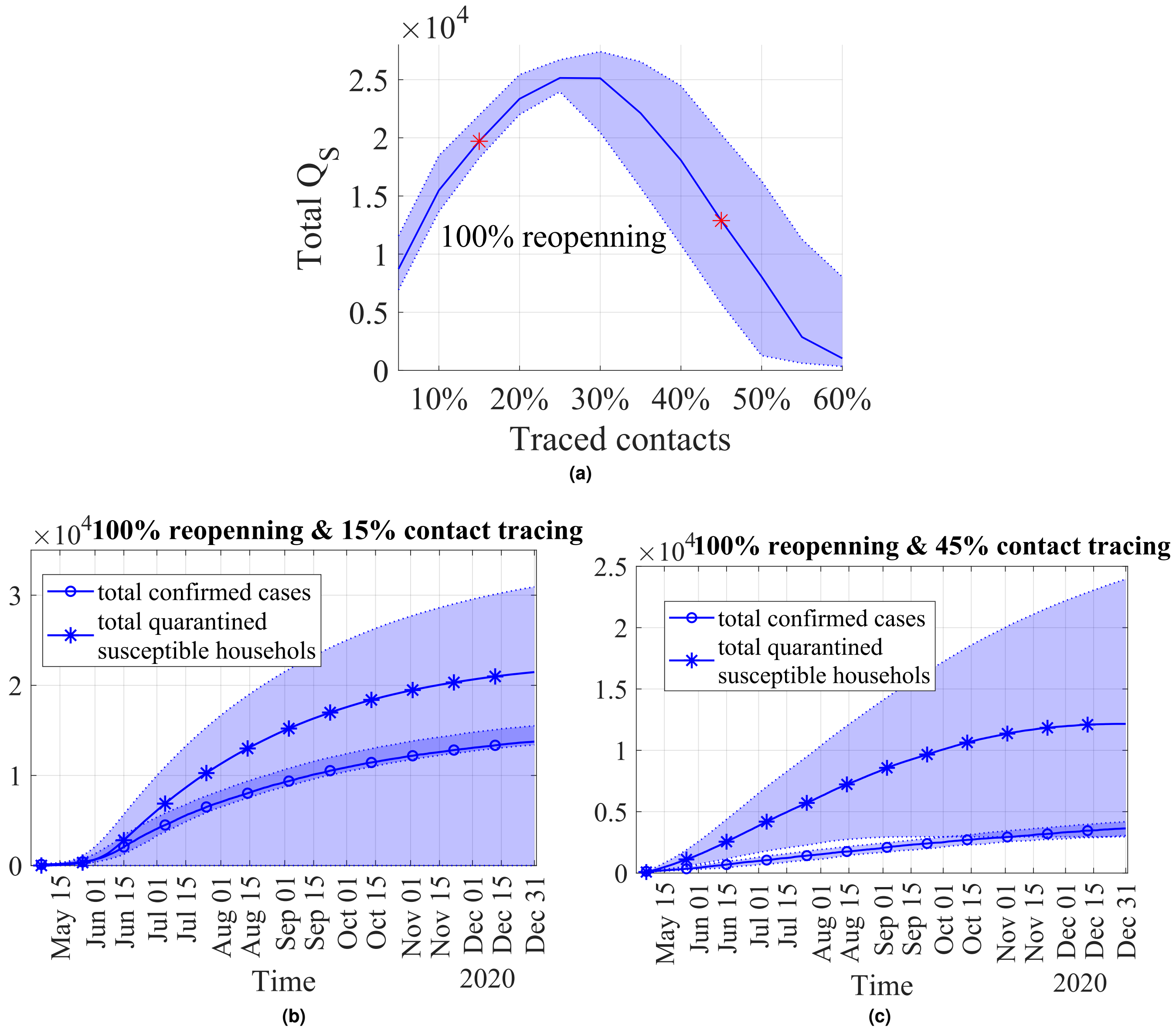

Figure S5. (a) The total number of quarantined susceptible households in eight months after May 4, 2020, for the SEICQ1 epidemic model for the 100% reopening scenarios with different tracing levels. (b) The total confirmed cases and the total quarantined susceptible households with time for the 15% contact tracing (for the first red star in the sub-figure (a)). (c) The total confirmed cases and the total quarantined susceptible households with time for the 45% contact tracing (for the second red star in the sub-figure (a)) This figure is showing the median (solid lines) and interquartile range (shaded regions) of 1000 stochastic realizations.
